# Targeted Delivery Methods for Anticancer Drugs

**DOI:** 10.3390/cancers14030622

**Published:** 2022-01-26

**Authors:** Valery V. Veselov, Alexander E. Nosyrev, László Jicsinszky, Renad N. Alyautdin, Giancarlo Cravotto

**Affiliations:** 1 Center of Bioanalytical Investigation and Molecular Design, Sechenov First Moscow State Medical University, 8 Trubetskaya ul, 119991 Moscow, Russia; sacrednibelung@yandex.ru (V.V.V.); rerik2050@mail.ru (A.E.N.); 2 Department of Drug Science and Technology, University of Turin, Via P. Giuria 9, 10125 Turin, Italy; ljicsinszky@gmail.com; 3 Department of Pharmacology, Sechenov First Moscow State Medical University, 119991 Moscow, Russia; alyautdin@expmed.ru; 4 World-Class Research Center “Digital Biodesign and Personalized Healthcare”, Sechenov First Moscow State Medical University, 8 Trubetskaya ul, 119991 Moscow, Russia

**Keywords:** nanocarriers, metal nanoparticles, dendrimers, oligo- and polysaccharides, solid-lipid systems, stimuli-responsive

## Abstract

**Simple Summary:**

The current main technological strategies for the delivery of anticancer drugs are discussed herein. This comprehensive review may help researchers design suitable delivery systems.

**Abstract:**

Several drug-delivery systems have been reported on and often successfully applied in cancer therapy. Cell-targeted delivery can reduce the overall toxicity of cytotoxic drugs and increase their effectiveness and selectivity. Besides traditional liposomal and micellar formulations, various nanocarrier systems have recently become the focus of developmental interest. This review discusses the preparation and targeting techniques as well as the properties of several liposome-, micelle-, solid-lipid nanoparticle-, dendrimer-, gold-, and magnetic-nanoparticle-based delivery systems. Approaches for targeted drug delivery and systems for drug release under a range of stimuli are also discussed.

## 1. Introduction

Over the past 30 years, the number of successful cancer treatments has significantly increased, predominantly driven by our improved understanding of carcinogenesis processes, cell biology, and the tumor microenvironment [1,2]. However, many cancers are still fatal despite the sustained effort being invested in preclinical and clinical research. One of the ways to improve the survival rate of cancer patients is the targeted delivery of anticancer drugs. Advances in biomedical science and biotechnology have led to the discovery and development of effective drug carriers such as liposomes, dendrimers, and gold and magnetic nanoparticles [3,4,5,6]. The principal difference between these new types of formulation and classical ones is their suitability for the potential development of technologies for targeted drug delivery to specific tissues, cells, and even intracellular organelles. The essence of targeted delivery lies in the surface of a drug container (carrier) bearing a modified drug or molecule with a functional group that can be recognized by the target cell receptors. Folic acid modification is a classic example as it is actively taken up by tumor cells [7,8,9]. Antibodies and aptamers are universal molecules that recognize the surface of a target cell [10,11,12]. Thanks to advances in basic biomedical research, the antigenic portraits of cells are becoming more and more detailed, allowing us to distinguish one cell from another based on their surface characteristics. Orally or parenterally administered medicines are distributed throughout the body, with only a small portion reaching the target area. Targeted delivery methods, therefore, make it possible to reduce the dosage of an administered drug and minimize its effect on other cells, which is very important in chemotherapy as drugs are highly toxic. The presence of recognizing or recognizable molecules on the surface of a delivery system allows it to concentrate on the desired area. It is also vital that the delivery system penetrates the cell and that the drug is then delivered to the nucleus, mitochondria, endoplasmic reticulum, and other organelles. In fact, the concept of intracellular drug delivery is under active development. Knowledge of the signaling pathways involving proteins that lead to different cellular structures is essential to achieve efficient intracellular transport [13]. Equally important is the need for more knowledge of the motor proteins of cells, which directionally move loads over long distances inside cells. It is also necessary to understand the mechanisms by which drugs are released from delivery systems, including diffusion, degradation, swelling, and other processes that can control the release of drugs [14,15,16].

## 2. Types of Containers and Carriers

A drug-delivery system may be considered suitable for clinical practice if it is non-toxic, biocompatible, stable in blood, non-immunogenic, non-thrombogenic, and biodegradable [17].

The enhanced permeability and retention (EPR) effect is the principle behind the passive targeting used in all containers and carriers. This principle and term were proposed in 1986 [18]. Rapid tumor growth is accompanied by neovascularization with wide fenestrations and the suppression of lymphatic drainage [19]. Traditionally, the EPR concept presumes that small molecules enter via diffusion and leave the interstitial space of the tumor, whereas macromolecules (containers, carriers) are no longer able to do so after extravasation [20]. In addition to the traditional explanation, other theories concerning how the pathophysiological characteristics of tumor growth shape the EPR effect have been put forward [21,22]. Thus, the EPR effect has been accepted as a universal principle incorporated into the design of anticancer drug-delivery systems [23]. However, there are currently serious disputes about the effectiveness of the EPR effect when using nanoparticles [24,25]. At the same time, it is important to note that the current understanding of the EPR effect is based on results obtained in animal models, meaning that the results of EPR-effect studies in patients must be collated if delivery systems that fully exploit the EPR effect are to be successfully designed [26].

### 2.1. Liposomes

Liposomes are spherical vesicles consisting of one or more lipid bilayers. A liposome has a hollow structure that is usually filled with a solvent and can deliver a variety of substances. Its hydrophobic membrane allows it to merge with cell membranes and transport its contents inside cells. Liposomes are most often composed of phospholipids and cholesterol, but may also include other lipids to improve endocytosis and tissue compatibility. Many methods have been developed to produce a range of liposomal compositions [27], and all described liposome fabrication methods combine lipids with the aqueous phase in some way [28,29]. The thin-layer hydration method, also known as the Bangham method, is one of the first and still most commonly used methods for the preparation of liposomes [30]. This method involves lipids being dissolved in the organic phase and removing the organic solvent, usually by evaporation, to form a lipid film. The lipid film is then dispersed in an aqueous medium that contains the drug under vigorous stirring to form the sealed spherical structures; liposomes. The short elimination half-life of liposomes, caused by their opsonization principally in the liver and spleen, is a crucial weak point in their use [31]. The modification of liposome surfaces with various functional ligands, such as polyethylene glycol (PEG) coating, reduces the interaction between the surface and blood components, thus ensuring that the liposomes have a longer residence time in the bloodstream [32]. PEG can be attached to liposome surfaces in a variety of ways:

- Physical adsorption onto the surface of liposomes.
- Covalent attachment using reactive groups on the surface of preformed liposomes.
- Inclusion of a PEG-lipid conjugate in liposome preparations.

The most common method anchors the polymer in the membrane using a cross-linked lipid (e.g., PEG-distearoylphosphatidylethanolamine) [33]. The presence of PEG on liposome surfaces reduces their aggregation [34]. To ensure targeted delivery, PEG is also often covalently bound to proteins (transport, signaling) so that, while the mechanism of action of the proteins does not change, there is a change in protein pharmacokinetics; PEG-asparaginase (used in the treatment of leukemia), PEG-aldesleukin (an antineoplastic agent), PEG-filgrastim (for the treatment of chemotherapy-induced febrile neutropenia), and PEG-epoetin-β (for the treatment of anemia) are commonly used in the treatment of cancer [35,36]. The liposomal delivery of anticancer drugs has been successfully used in cancer therapy for several decades [37].

### 2.2. Micelles

Micelles are particles of tens of nanometers in size with a hydrophobic core and a hydrophilic surface and are commonly used as carriers of hydrophobic drugs. Like liposomes, they can be delivered directly into the bloodstream through the respiratory tract or skin. In recent years, amphiphilic block copolymers, which spontaneously form micellar structures, have attracted much attention because of their use in the delivery of cytostatic drugs [38,39]. Amphiphilic block copolymers are usually assembled from two or three blocks, with PEG being the most common hydrophilic block in the copolymer structure. Other hydrophilic block-forming polymers include chitosan, polyvinylpyrrolidone, and poly(N-isopropyl acrylamide) [40]. Polymers of various compound classes are used as hydrophobic polymer blocks for micellar core creation: polyethers (poly(propylene oxide)) polyesters (polylactide), polycarboxylic acids (poly(aspartic acid)) and lipids (distearoylphosphatidyl ethanolamine) [40]. Micelles that contain functional groups (-NH2, -COOH) in their core can transfer drugs by chemical modification and not just by physical encapsulation [41], and various cytostatic drug micelles (doxorubicin, paclitaxel) have shown significant results in several in-vitro and in-vivo studies [42]. Paclitaxel encapsulated in micelles has been tested in clinical trials in patients with malignant tumors with a resulting reduction in toxicity and no change in the antitumor activity compared to free paclitaxel [43].

### 2.3. Solid-Lipid Nanoparticles

Solid-lipid nanoparticles are colloidal nanoparticles stabilized by surfactants and composed of mono-, di- and triglycerides, solid fats, and waxes. They have been developed as an alternative to liposome technologies to increase stability, modulate the release of encapsulated drugs, reduce costs, and simplify manufacturing [44]. Unlike liposomes, which are usually injected into the body intravenously, intraperitoneally, subcutaneously, and orally, solid-lipid nanoparticles can be administered via different routes, via inhalation, intranasally, and intravesically [45], thus ensuring the local targeting of the drug. Recent in-vitro and in-vivo experiments have shown that solid-lipid nanoparticles that contain cytostatic drugs appear to be superior to conventional drug solutions and are comparable to other encapsulated systems in many aspects, such as efficacy, pharmacokinetics, and bioavailability [46]. However, clinical studies have not yet been conducted in this area.

### 2.4. Gold Nanoparticles

Gold nanoparticles (AuNP) can boast a combination of unique physical and chemical properties relative to other biomedical nanotechnologies and can selectively deliver cytostatic drugs [47,48]. AuNPs offer significant potential for new approaches to cancer treatment as they are easy to produce, have low toxicity, and display antiangiogenic properties [49]. AuNPs are up to 100 nm in size, have a pronounced EPR-effect, and, as a result, preferentially accumulate in tumors.

AuNP-based supports are most often synthesized using colloidal methods; gold salts (e.g., hydrogen tetrachloroaurate (III)) are reduced in the presence of surface stabilizers that prevent the aggregation of the resulting solution [50]. Spherical AuNPs are principally used to create delivery systems because they can be synthesized on a large scale with high monodispersity. The other forms of AuNP include nanorods, nanoshells, and nano cells [51]. AuNPs can undergo surface modification thanks to their covalent and non-covalent bond-forming properties [51]. A stabilizing agent (e.g., citric acid) is responsible for the overall charge of the AuNP surface. The correct choice of a stabilizing agent allows various biomolecules (DNA, antibodies, polypeptides) to be conjugated to the AuNP surface via electrostatic interactions, whereas covalent attachment to AuNPs is usually achieved via the interaction between gold and thiol, amine, and carboxylate functional groups [52]. Unlike liposomes and micelles, the drug is conjugated directly to the AuNP surface using various linkers [52,53,54]. It is worth noting that the overwhelming majority of studies on AuNP-based directional transport are based on spherical AuNPs, and this is, at least in part, because they undergo surface modification and penetrate cells more easily than more complex AuNPs. A drug conjugated to AuNPs has shown increased antitumor potential compared to the free drug in in-vitro and in-vivo studies [47,55,56,57,58].

### 2.5. Magnetic Nanoparticles

Magnetic targeting is of great interest in the treatment of malign tumors as the technique not only provides targeted drug delivery but also makes it possible to monitor the accumulation of magnetic nanoparticles (MNP) in tumors using magnetic resonance imaging (MRI) [59,60]. MNPs that carry a drug are first accumulated in the target tissue using an external magnetic field, and the drug is then released from the MNPs in a controlled manner [61].

MNPs are magnetic materials with small particle sizes (from 10 to 100 nm), a large specific surface area, magnetic response, and superparamagnetism [62]. This superparamagnetism means that MNPs are in a single-domain state, as they are uniformly magnetized throughout the entire volume [63], and that the orientation of their magnetic moment changes with temperature [63]. Iron oxides, for example magnetite (Fe_3_O_4_ or FeO.Fe_2_O_3_) and maghemite (γ-Fe_2_O_3_), are usually used for MNP production [60,64]. The MNP core, which consists of magnetite, maghemite, or a mixture of the two, is usually obtained via the precipitation of Fe^2+^ and Fe^3+^ iron salts from an aqueous solution [65,66]. Moreover, it is possible to regulate the size of the resulting nanoparticles by adding various iron salts (chloride, sulfate, nitrate, etc.) and by changing the ratio of Fe^2+^ and Fe^3+^, the pH, and the ionic strength in the solution [62,67]. Reactions are carried out in an inert atmosphere to prevent the oxidation of the formed nanoparticles [68]. The formed MNPs have a hydrophobic surface and are coated with synthetic and natural polymers to reduce nanoparticle agglomeration [60] and further modify the surface to conjugate drugs and biomolecules [69]. The most commonly used polymers are PEG, dextran, polyvinylpyrrolidone, polyaniline, alginate various fatty acids, and chitosan [70,71]. In general, the conjugates of MNPs with various cytostatics show decreased overall toxicity, and the concentration of cytostatic agents is required to achieve a therapeutic effect [72,73,74,75,76,77]. The ability of MNPs to accumulate in tumors has also been confirmed by MRI [59,74,78,79,80].

### 2.6. Dendrimers

Dendrimers are three-dimensional, monomolecular, highly branched monodisperse macromolecules [81] that usually have rotational symmetry and often take on a spherical shape. In general, dendrimers have a hydrophobic core from which they branch, ending in terminal functional groups responsible for their solubility in water [82]. These dendrimers can retain hydrophobic drugs and increase their concentration in water. Biocompatibility, easy excretion from the body, and a significantly improved EPR effect are the most remarkable advantages of dendrimers. However, dendrimers have one significant drawback; they are cytotoxic for normal cells due to the physiological stability of cationic groups on their surfaces [83]. The problem of dendrimer cytotoxicity is usually solved by modifying their surface using biocompatible polymers, for example, PEG. The PEG-modified dendrimer surface provides the necessary screening of the cationic surface charge, which leads to a biologically safe carrier [84].

Dendrimer synthesis is a rather laborious process. There are two principal approaches to the synthesis of dendrimers; divergent and convergent methods [85]. In the divergent version, a base reagent (a molecule that is protected at its end groups, if necessary) is attached to the original branching center (which has several end groups). The protecting groups are removed, and a 1st generation dendrimer is formed. Subsequently, dendrimers of higher generations are obtained by attaching either the original branching center or the base reagent, followed by deprotection [86]. In the convergent method, the arms of the dendrimer are synthesized first and then connected [86], and this method produces more monodisperse dendrimers than the divergent version. However, the size of dendrimers obtained using the convergent method is limited due to steric hindrance, whereas dendrimers of a wider variety of sizes can be obtained using the divergent method [85,86]. The most widely used dendrimers are currently the commercially available poly(amidoamine) (PAMAM) dendrimers [87,88,89]. Delivery systems based on poly(propylene imine) [90], polylysine [91], carbosilane [92], and phosphorus dendrimers [93] have also been developed. Numerous studies have shown the effectiveness of using different dendrimers for targeted transport in cancer therapy [94,95,96,97,98,99], and several clinical trials using various dendrimers as targeted delivery systems are underway [100].

### 2.7. Albumin-Based Nanoparticles

Albumin is the most abundant plasma protein in human blood, with a molecular weight of about 67 kDa. Due to its endogenous origin, it is non-toxic, non-immunogenic, biocompatible, and biodegradable [101]. Human serum albumin (HSA) and the cheaper bovine serum albumin (BSA) and ovalbumin (OVA) have been used to create delivery systems [102]. HSA has several ligand binding sites that can be used for transfer via both hydrophobic and electrostatic interactions [103,104], and the presence of a free cysteine residue on its surface means that albumin easily conjugates with a variety of ligands [105,106]. Receptors, such as albondin (Gp60) and secreted protein acidic and rich in cysteine (SPARC), have been shown to overexpress in some cancers [107] and can mediate albumin transcytosis [108], while the Gp30 and Gp18 receptors, the megalin/cubilin complex and the neonatal Fc receptor (FcRn) are also involved in albumin transport [106]. Albumin-based delivery systems can therefore accumulate in tumors via mechanisms beyond the EPR effect. Albumin-based nanoparticles are obtained by various methods, including emulsification, self-assembly of thermal gelation, desolvation, and nanospray drying [109,110]. The patented nanoparticle albumin-bound (NAB) technology, which consists of the evaporation of an emulsion with the creation of cross-links between albumin units, is the best-known preparation method for albumin-based nanoparticles [105,106]. In addition to Abraxane^®^, which is created with the help of NAB technology and has been successfully used in clinical practice [111,112,113], work is also underway to create a range of albumin-based nanoparticles [104,105,106,107,108,109,110,111,112,113,114,115,116,117,118].

### 2.8. Porous Materials

Zeolites are hydrated crystalline aluminosilicates consisting of tetrahedral groups, [SiO_4_]^4−^ and [AlO_4_]^5−^, united by common vertices into a three-dimensional framework. The open frame-cavity structure of zeolites has a negative charge, which is compensated for by counterions [119]. Zeolites have a porous structure that can absorb various substances, making zeolites an ideal material for drug-delivery systems [120]. To prevent the untimely release of a drug, either a zeolite with an optimal pore size is selected [121], or its surface is modified with various ligands [122,123]. In general, zeolites are promising carriers for creating systems for the delivery of cytotoxic substances [124,125,126,127].

Mesoporous silica particles (MSP) are another porous material used for drug delivery [128]. Their pore size can be adjusted from 2 to 50 nm, as in the case of zeolites, to tune them for a specific drug [129,130,131]. The surfaces of MSPs are rich in reactive silanol groups, which can be used for conjugation with various substances [132], and MSPs have been developed with several structures. The morphology and size of both the particles themselves and their pores can be controlled via the choice of a synthetic method [133,134]. MSP-based delivery systems have shown high drug-loading capacity, successfully controlled release, and increased antitumor activity [79,135,136,137].

### 2.9. Carbon Nanoparticles

Carbon has many allotropic modifications, including carbon nanotubes, fullerenes, and nanodiamonds, which have found applications as carriers for drug delivery [138]. Carbon-based quantum dots are also used (see the Section 2.10). Carbon nanoparticles have a high specific surface area and hydrophobicity. Carbon nanotubes and fullerenes have cavities in their structure and can encapsulate active substances [138,139]. However, unlike fullerenes and carbon nanotubes, the surface of nanodiamonds is rougher, which increases adhesion with drugs [140]. Under the action of acidic oxidation, carboxyl groups are formed on the surface of carbon nanoparticles and are used for surface modification, as well as for the covalent attachment of anticancer drugs [141,142,143]. Carbon nanoparticles with the desired properties can be obtained by correctly choosing and adapting the synthesis method [144,145,146].

While carbon nanoparticles are currently widely used for drug delivery, their toxic properties are concentration-dependent [147,148,149]. Attention should therefore be paid to the delivery method when developing carbon-nanoparticle supports. For example, it has been shown that the absorption of fullerene by the respiratory and digestive tracts is low [150]. In addition, as has been demonstrated, inhaled carbon nanotubes can act on the body similarly to asbestos [151].

### 2.10. Quantum Dots

Quantum dots are inorganic semiconductor nanocrystals and are typically up to 10 nm in size. Quantum dots have fluorescent, optical and electronic properties [152], with cadmium-compound-based and carbon quantum dots being the most widespread [153,154]. In addition to drug delivery, quantum dots can visualize cancer cells due to their unique optical properties, which derive from quantum and other effects [155]. The quantum dots used in biomedicine typically consist of a core and a coating with the core imparting optical properties to the system and the coating performing a protective function, which enables the surface to be functionalized with various ligands and is responsible for water solubility [156]. The quantum-dot core may be composed of cadmium compounds, such as cadmium selenide (CdSe), cadmium sulfide (CdS), and cadmium telluride (CdTe), and these quantum dots have shown notable results as drug carriers [157,158,159]. It is important to mention that these quantum dots are not biodegradable and are not cell and environmentally friendly due to the toxicity of cadmium compounds [160].

Carbon-based quantum dots, which can be classified as either carbon quantum dots or graphene quantum dots, are widely used in various fields of biomedicine [161]. They possess low toxicity, high specific surface area, high photostability and are easily modified [162]. Carbon-based quantum dots are excellent carriers for anticancer drugs due to their biocompatibility, ease of manufacture, and lower environmental impact [163,164,165,166].

### 2.11. Calcium Phosphate

Calcium phosphate (CaP)-based nanoparticles are crystalline formations of predominantly carbonate apatite capable of transporting a drug both on their surface and within their structure [167,168]. Minerals based on CaPs are the main inorganic components of the bones and teeth of vertebrates and humans [169]. CaP-based nanoparticles have several peculiar properties that make them attractive for delivering anticancer drugs. CaPs are fully biodegradable, release non-toxic calcium and phosphate ions upon degradation, and decompose faster than other inorganic nanoparticles (zeolites, mesoporous silica particles, carbon nanoparticles, and quantum dots) [168,170]. Moreover, CaP-based nanoparticles have pH-sensitive solubility; they are insoluble at the physiological pH of blood plasma (7.4) but quickly dissolve in acidic biological media (pH < 5), for example, in endosomes and lysosomes, where they rapidly release encapsulated substances [170,171,172]. There are currently many approaches for synthesizing CaP-based nanoparticles, and the careful selection of synthesis conditions makes it possible to control the size and morphology of the resulting particles [173,174,175,176]. Although nanoparticles with different morphologies are used for delivery in cancer therapy, including rod shapes [169,177], porous structures [178,179], and core-shell shapes [180,181], spherical nanoparticles are the most commonly used since they are more thermodynamically stable [182,183].

### 2.12. Oligo- and Polysaccharide-Based Drug-Delivery Systems

#### 2.12.1. Chitosan

**Figure 1:**
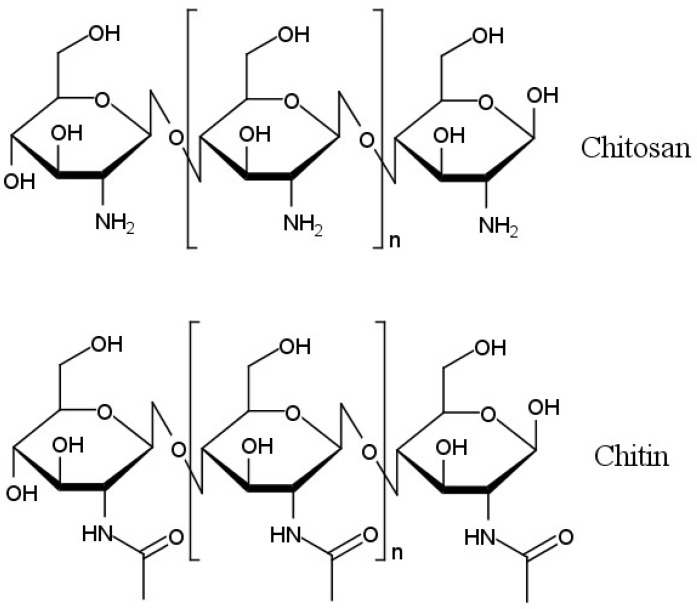
Structural formulas of chitosan and chitin.

Chitosan is a type of amino polysaccharide polymer (see Figure 1) produced via the deacetylation of chitin, and is the second most common biopolymer in nature after cellulose. Chitin is the main component of the exoskeleton of arthropods and many other invertebrates and is also part of the cell walls of fungi [184]. Chitosan has amino functionalities that are useful for biopolymer modification [185]. Its biodegradability, biocompatibility, low immunogenicity, and non-toxicity mean that chitosan is used in delivery systems for various chemotherapeutic drugs [186]. Chitosan and its derivatives, such as carboxymethyl chitosan, sulfated chitosan, sulfated benzaldehyde chitosan, and polypyrrole-chitosan, have been shown to have anticancer activity in and of themselves [187,188,189,190]. This property is assumed to be related to the antioxidant properties of chitosan and its derivatives, which are capable of trapping cancer-causing free radicals [191].

As mentioned earlier, chitosan can be a hydrophilic moiety in an amphiphilic block copolymer [192]. The presence of free amino groups in the chitosan backbone grants it a unique polycationic character that ensures that negatively charged drugs, such as doxorubicin, are properly encapsulated [193]. Some chitosan-based hydrogels that contain a significant amount of water and retain a self-organized three-dimensional structure have been developed and can be used for the encapsulation and delivery of anticancer drugs [194,195]. Various forms of delivery systems, such as microspheres, film capsules, etc., have been obtained using water-insoluble species of chitosan [196], meaning that the properties of chitosan-based delivery systems are easy to modulate. Depending on the preparation method selected, it is possible to regulate the particle size, toxicity, thermal and chemical stability, and release kinetics [197].

#### 2.12.2. Cyclodextrins

**Figure 2:**
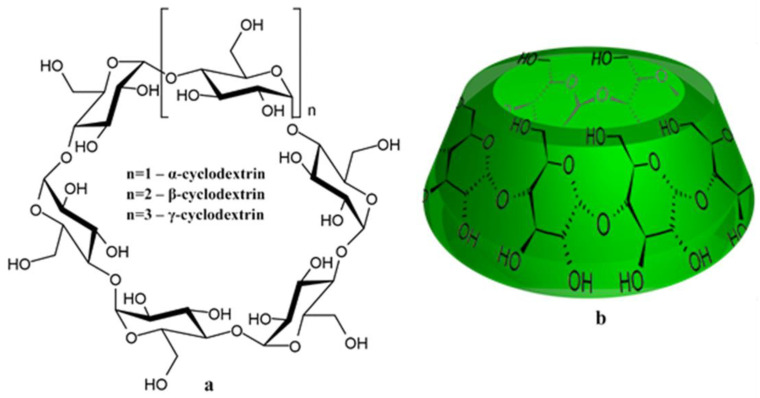
(**a**) Structural formula of cyclodextrins; (**b**) 3D structure of cyclodextrins.

Cyclodextrins (CDs) are a family of cyclic oligosaccharides that consist of glucose subunits obtained by enzymatic means from starch [198]. The most commonly used CD types are α-, β- and γ-CDs (Figure 2a), named according to the number of glucose residues they possess [199,200].

CDs have a hydrophilic outer surface and a significantly less hydrophilic cavity. They take the form of a truncated cone with a cylindrical-torus cavity inside (Figure 2b) [201]. CDs can form various complexes with hydrophilic, lipophilic, and amphiphilic substances. CDs, therefore, can often increase the solubility and bioavailability of many anticancer drugs [202], and various cyclodextrin derivatives are widely used to create drug-delivery systems [203].

CDs are often combined with other nanoparticles to create delivery systems [201]. It has been shown that the loading of liposomes with anticancer drugs in combination with cyclodextrin increases their half-life, reduces toxicity, and increases liposome loading [204,205,206,207]. Several CD-based polymers have also been developed and used successfully in drug transport [208].

The cross-linking of CDs results in unique particles, namely CD polymers and nanosponges. Nanosponges are a type of nanoparticle that has a porous structure with a pore size of several nanometers. Due to their unique structure, CD polymers and nanosponges can encapsulate various substances in their pores and act as drug transporters [209]. One of the advantages of natural CD-based delivery systems is the creation of effective oral, mucosal and transdermal drug formulations [210]. Cyclodextrin-based macromolecules can transfer oligonucleotides, siRNAs, or their fragments into the cells. Though the promising results reported by these associations, experimental trials are still in progress [211,212,213,214].

#### 2.12.3. Pectins

Pectins are polysaccharides that are mainly formed from residues of galacturonic acid. Pectins are extracted in different ways from higher plants, mainly from their fruits. Consequently, the structures of pectins can be very diverse, although they can be classified into three types based on their general characteristics: homogalacturonan, rhamnogalacturonan-I, and substituted galacturonans [215]. Pectin and its various modifications have anticancer activities [216,217,218], and the majority of studies on natural and modified pectins, and their delivery systems, have mainly focused on colon cancer [219,220,221]. This is primarily because pectin is not digested in the gastrointestinal tract until it reaches the colon, where it is fermented and breaks down to release encapsulated active ingredients [222,223]. Pectin-based microgranules and microspheres have been developed to encapsulate anticancer drugs and release them directly into the colon [224,225]. Another use of pectins is as a drug carrier in the preparation of various hydrogels [226,227]. As pectins contain carboxyl groups, it is possible to use them to create negatively charged particles that retain drugs thanks to electrostatic interactions [228]. Moreover, pectin has been used to create self-organizing polymer nanoparticles to deliver ursolic acid [229].

## 3. The Targeting Methods of Delivery Systems

Active targeting is used to increase the concentration of cytostatics in the desired organ or tissues to achieve higher and more selective therapeutic activity. The surface of the container or carrier is modified with various recognizable or recognition molecules, such as monoclonal antibodies or their fragments, aptamers, proteins, peptides, and low molecular weight compounds, to grant active-targeting properties [230].

### 3.1. Antibodies and Aptamers

Numerous monoclonal antibodies (mAb) have recently been developed against various epitopes of cancer cells and are used as therapeutic agents in and of themselves [231]. mAb-conjugated containers or carriers specifically bind to a target cell (receptor, protein, etc.) in the desired areas and then release the encapsulated drug. The surface modification of the carrier systems with mAbs can either be achieved via non-covalent physical interactions or the formation of covalent bonds [232], with non-covalent bonding being faster than covalent. However, the antigen-binding domains of mAbs are arranged chaotically in non-covalent conjugation, which can lead to disruption in mAb functionality [233]. The most commonly used method for non-covalent conjugation is the streptavidin-biotin method [234]. It consists of the preliminary non-covalent conjugation of the surface of the delivery systems with streptavidin, which has a high affinity for biotin, which, in turn, is covalently linked to the mAbs.

The most commonly used cancer targets for mAbs are:

- Epidermal growth factor receptor (EGFR) [235,236,237].
- Human epidermal growth factor receptor 2 (HEP2) [238,239,240].
- B-lymphocyte antigen CD19 [241,242,243].
- Guanine deaminase (GAH) [244,245].
- Receptor cluster of differentiation 47 (CD47) [246,247].

Conjugation with aptamers is a newer approach to targeted delivery. Aptamers are oligonucleotides (DNA, RNA aptamers) or peptide molecules that specifically bind to specific target molecules and can be considered analogs of monoclonal antibodies. However, they have many advantages over antibodies. Their production is much easier, cheaper, and faster than monoclonal antibodies. They have a much smaller size and, therefore, more easily penetrate tissues and cells, as well as having higher affinity and specificity [248]. The potential for the in-vivo targeting of RNA aptamers in cancer therapy was demonstrated for the first time in 2006 [249]. More than 20 different systems for targeted transport are currently being developed using oligonucleotide aptamers [250].

Peptide aptamers consist of a short (10–20 amino acid), conformationally limited peptide sequence that is inserted into a scaffold protein (most often the bacterial protein thioredoxin A) [251,252]. A unique feature of peptide aptamers is that their variable region has a double limitation as both ends are connected to a framework (protein), unlike oligonucleotide aptamers and antibodies [252]. For this reason, peptide aptamers have limited conformations and require less energy to bind to the target, which, in turn, increases their affinity.

### 3.2. Proteins and Peptides

**Figure 3:**
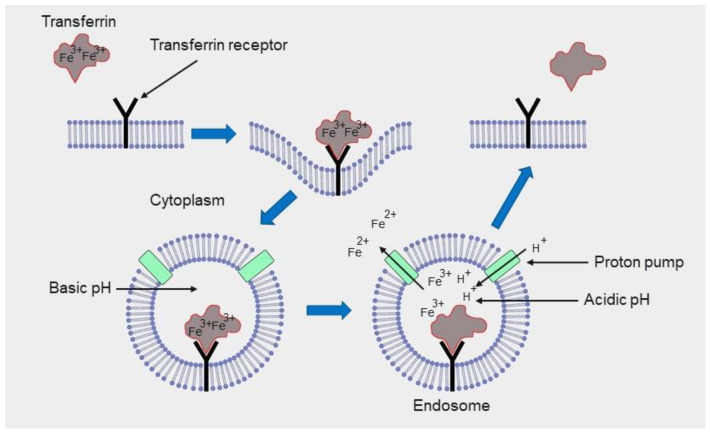
Schematic representation of the penetration of transferrin into a cell via receptor-mediated endocytosis. Original diagram inspired by [263].

The most commonly used protein for targeting delivery systems is transferrin, a serum glycoprotein that transports iron into cells by binding to transferrin receptors on the cell surface [253]. The transferrin receptor is present in malignant tumors at levels that are hundreds of times higher than in normal cells (Figure 3) [254]. Containers and carriers, their surface modified by transferrin molecules, can therefore penetrate cancer cells and accumulate in them [230]. The following proteins and peptides are used to target other receptors that are overexpressed in cancer cells:

- A designed ankyrin repeat protein (DARPin) can target the Epithelial cell adhesion molecule (EpCAM) [255,256].
- The peptide K237 targets the kinase insert domain receptor (KDR) [257,258].
- The peptide bombesin targets the gastrin-releasing peptide receptor [259,260].
- The peptide octreotide targets the somatostatin receptor type 2 [261,262].

### 3.3. Low Molecular Weight Compounds

Modifying the surfaces of containers and carriers with folic acid is currently a commonly used methodology to ensure the delivery of an encapsulated drug into cancer cells [264]. Folic acid is a low molecular weight compound, a vitamin required by eukaryotic cells for the biosynthesis of purines and pyrimidines [265]. Folate uptake by cells occurs via two mechanisms: via the low-affinity-reduced folate carrier (RFC), found in almost all cells, and via the high-affinity glycosylphosphatidylinositol-linked folate receptor (FOLR), which has limited distribution [266]. FOLR can transport conjugated folate into cells, unlike RFC [266,267], and FOLR is also significantly expressed in different types of human tumors but is minimally expressed in most normal tissues [268]. Consequently, FOLR is a target for the selective delivery of anticancer molecules. A wide range of containers and carriers, such as liposomes, micelles, gold nanoparticles, dendrimers, and magnetic nanoparticles, have been targeted to cancerous tumors using folic acid as the targeting component [269].

Other small molecule compounds are used to target specific cancers. For example, the asialoglycoprotein receptor (ASGPR) is overexpressed on the surfaces of hepatocytes in hepatocellular carcinoma [270]. Studies have shown that modifying the surface of a container or carrier with D-galactose residues or N-acetylgalactosamine effectively targets the delivery system to hepatocytes via ASGPR [271,272]. Surface modification with lactose is also used to target hepatocytes via ASGPR [273,274]. It has been shown that the cells of some cancers, such as brain cancer, colon cancer, melanoma, and breast cancer, overexpress sigma receptors [275,276,277,278]. The conjugation of containers and carriers with anisamide, which has a high affinity for sigma receptors, has been proposed as a means of targeting sigma receptors [279]. More than ten different delivery systems that use the sigma receptor as a target have already been developed [280].

### 3.4. Small Molecule-Drug Conjugates

Small molecule-drug conjugates (SMDC) are a drug delivery system without using nanocontainers and nanocarriers [281]. Typically, SMDCs consist of an anticancer agent coupled to a targeting ligand via a linker capable of being cleaved under various stimuli (See Section 4 for details) [282]. Besides the binding ability to the cellular target, the spacer also increases the hydrophilicity of the conjugate [281,283]. Antibodies [284,285]) and aptamers [286], peptides [287,288] and low molecular weight compounds [289,290] can act as a targeting ligand. Although SMDCs do not exhibit an EPR effect and, therefore, do not passively accumulate in solid tumors, they nevertheless passively perfuse the cancer mass more thoroughly and faster than nanoparticles [282]. When creating SMDCs, it should be taken into account that they have a short half-life compared to nanoparticles [291]. Various SMDCs have been developed and are being used successfully in cancer therapy [292].

Various small interfering RNA (siRNA) conjugates are also used for cancer gene therapy [293,294]. Chemical modification of siRNAs (at the 2′ position, at the ribose ring, or using nucleotide phosphorothioate) improves their stability, increases cell specificity, and reduces off-target effects [295]. siRNA conjugates show efficient RNA interference both in vitro and in vivo [296].

## 4. Stimuli-Responsive Drug Release

To be delivered to the desired area of the body, an active substance is either encapsulated in the delivery system or covalently associated with it. There are two main mechanisms of drug release: firstly, as a result of endocytosis or fusion with the cell membrane (in the case of lipid delivery systems), and, secondly, under the influence of stimuli [297]. These stimuli can be internal and thus inherent to the affected area of the body, such as changes in enzyme levels, pH, and temperature; or external, such as a magnetic field, ultrasound, and light [298].

### 4.1. Enzyme-Sensitive Release

The expression pattern of enzymatic proteins in the tumor may be altered in some types of cancer [299]. There are two main approaches to controlling the release of a drug from delivery systems under the action of enzymes [300]:

- The drug is conjugated to the delivery system with a linker cleaved by an enzyme that is overexpressed in the tumor environment.
- Enzyme cleavage sites are embedded into the envelope of the scaffolds, thereby destroying the envelope near or inside the tumor and releasing the encapsulated drug.

Several materials sensitive to various enzymes have been obtained to date [300]. For example, an octapeptide sensitive to metalloproteinase has been developed and used as a linker [301]. Other enzymes that have been used for drug release include phospholipase [302], α-amylase [303], glucose oxidase [304], and cancer-associated proteases [305].

### 4.2. pH-Sensitive Release

Due to changes in the metabolic environment, the extracellular pH is usually lower in tumors (≈6.5) than in blood and normal tissues (≈7.4) [306]. The pH level in tumor tissue is not uniform; intracellular pH is similar in tumor and normal tissues, and extracellular pH is more acidic [307]. This difference in pH means that a cellular transmembrane gradient is formed between normal tissue and tumor tissue. Exploiting this gradient allows drugs to be directly delivered into the cytosol of cancer cells, which are weak electrolytes with the corresponding pKa [307]. A weakly acidic drug in protonated form can freely penetrate through a cell membrane, reach a region with a more basic pH, and then become trapped inside the cell, leading to a significant difference in drug concentration between normal and tumor tissues.

There are two main approaches to using pH as a stimulus for drug release. The first approach is to introduce various chemical bonds, which are hydrolyzed and destroyed under conditions of acidic pH, into the delivery system. Most often, bonds are introduced into the delivery system of drugs, as presented in Table 1.

The second approach exploits the ability of different polymers to be protonated/deprotonated at different pH levels. At physiological pH, such polymers remain deprotonated/deionized, but under acidic conditions, the polymers are protonated or change their charge, causing structural transformation or disintegration in the delivery system and the subsequent release of the encapsulated drugs [16]. Conjugation of urocanic acid with various polymers makes it possible to give them pH-dependent properties [308,309].

**Table 1:** List of pH-labile bonds.

Acid Labile Bond	Transformation Scheme	Ref.
Hydrazone	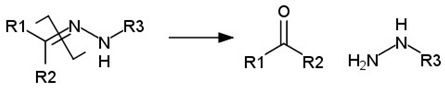	[310,311]
Oxime	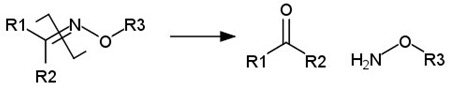	[312]
Imine	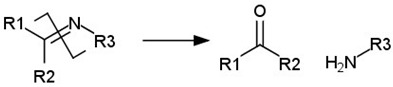	[313,314]
Acetal/ketal	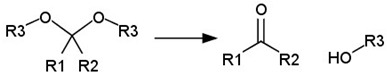	[315,316]
Orthoester	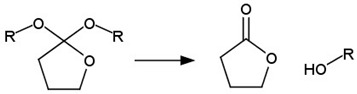	[317]
Amide	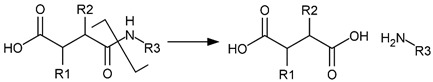	[318]

### 4.3. Temperature-Sensitive Release

Mild hyperthermia plays a pivotal role in changing the tumor microenvironment by increasing blood-flow velocity, oxygenation, and vascular permeability [319]. It has been shown that most delivery systems, up to 400 nm in diameter, can extravasate from the tumor environment into tumor cells when heated to 42 °C in vitro [320]. Moreover, the inclusion of thermosensitive fragments in a delivery system changes their properties in areas with elevated temperatures, leading to the release of the encapsulated drug. At a specific temperature, lipid carrier systems that contain lysolipids or oligoglycerol undergo a gel-liquid phase transition involving the release of the active substance [321]. Several thermosensitive polymers have been developed with a lower critical solution temperature (LCST) of about 40 °C. Below this temperature, the polymers are soluble in water but become insoluble in water above this temperature. Such polymers are used in anticancer-molecule delivery systems [320]. Table 2 lists some characteristic polymers used for temperature-sensitive release from delivery systems and indicates their LCST [322]. Hyperthermia in the environment of a tumor can also be caused externally, for example, by applying an alternating magnetic field around the tumor, causing magnetic nanoparticles to heat up and creating hyperthermia in the area. Furthermore, in an alternating magnetic field, the magnetic nanoparticles themselves have a strong cytotoxic effect on cancer cells [239,323]. The creation of hyperthermia in the desired area can also be achieved using a laser; photothermal inducing agents can be included in the structure of delivery systems and absorb emitted light and convert it into local heat [324]. The most commonly used photothermal material is gold nanoparticles [325,326].

### 4.4. Other Stimuli

The redox environment of tumor cells is changed by an increased level of glutathione (GSH) usually 4 times higher than in normal cells [331]. Glutathione regulates the reducing environment of the cell by forming and destroying disulfide bonds via reaction with the excess of reactive oxygen species (ROS) [332,333]. Redox-sensitive delivery systems usually contain disulfide, diselenide, or succinimide-thioether bonds [334,335,336,337]. Under the influence of glutathione, these bonds are reduced and destroyed (Table 3).

Some studies have shown, indicating oncogenic transformation compared to normal cells, that cancer cells constantly generate high levels of intracellular ROS, such as hydrogen peroxide, hydroxyl radical, and superoxide anion [338]. Some ROS-sensitive transport systems have also been developed to exploit this abnormal biochemical change [339,340], and all of the delivery systems that have been developed contain ROS-sensitive linkers. In essence, the linkers are based on the organic compounds of chalcogens (S, Se, Te), such as thioesters [341,342], thioketals [343], diselenides [344], monoselenides [345], and tellurides [346]. Under the action of ROS, the two-stage oxidation of thioesters, monoselenides, and tellurides occurs, first to the oxidation state of +4 and then to +6. Accordingly, delivery systems that contain these groups undergo a phase transition from hydrophobic compounds to more hydrophilic ones [347]. Linkers that contain other ROS-sensitive groups are oxidized with bond cleavage (Table 3). As in the case of pH-sensitive release, delivery systems with ROS-sensitive bonds release their drugs either via a phase transition or the breaking of a chemical bond.

It has been shown that monosulfides and monoselenides are only ROS-sensitive linkers despite disulfides and diselenides being redox- and ROS-sensitive linkers [348,349]. Arylboronic ethers are widely used as the ROS-sensitive linker as, under the action of hydrogen peroxide, arylboronic esters are oxidized to boronic acid and phenol, and the bond in the para-position of the aryl ring is destroyed [350].

**Table 3:** List of labile groups sensitive to GSH and ROS.

Labile Group	Transformation Scheme	Ref.
Disulfide	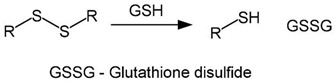	[336]
Succinimide-thioether	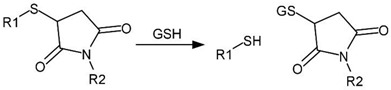	[337]
Organic compounds of chalcogens	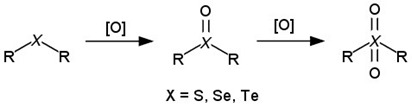	[342,345,346]
Diselenides	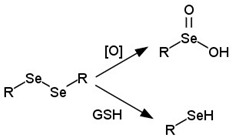	[344]
Thioketals	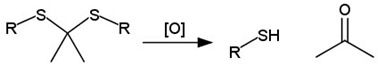	[343]
Arylboronic ethers	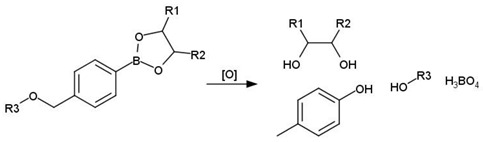	[350]

## 5. Conclusions

Over recent decades, tremendous progress has been made in the field of targeted delivery in cancer therapy. Several targeted-delivery drugs have been approved and included in clinical practice. Delivery systems can target different parts of a tumor using specific targeting fragments and avoid the problems associated with multidrug resistance. With detailed studies of the physiological differences between normal and diseased tissues, it is possible to develop target-specific drug delivery systems able to respond to local stimuli. However, some aspects require a more detailed study. In fact, a deeper understanding of the EPR effect, of the interactions between nanoparticles and cells, of tumor targeting, and of the metastatic microenvironment is certainly needed. Moreover, further insights into the biodistribution, pharmacokinetics, toxicity, and role of delivery systems in therapeutic protocols are essential if they are to become part of standard-treatment algorithms. Adverse immunological reactions also require careful consideration when using targeted delivery. Only once studies into these factors are complete will it be possible to unleash the full potential of cytostatic drug-delivery systems in cancer therapy.

## Figures and Tables

**Table 2:** The LCST of polymers in an aqueous solution.

Polymer	LCST, °C	Ref.
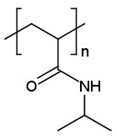 Poly(*N*-isopropylacrylamide), PNIPAAm	≈32	[327]
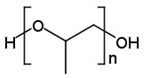 Polypropylene glycol, PPG	≈40	[328]
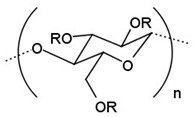 Carboxymethyl cellulose, CMC	40–50	[329]
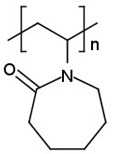 Poly(*N*-vinylcaprolactam)	≈38	[330]
